# Does spending on refugees make a difference? A cross-sectional study of the association between refugee program spending and health outcomes in 70 sites in 17 countries

**DOI:** 10.1186/s13031-016-0095-4

**Published:** 2016-12-07

**Authors:** Timothy M Tan, Paul Spiegel, Christopher Haskew, P Gregg Greenough

**Affiliations:** 1 Columbia University Mailman School of Public Health, 60 Haven Ave, Floor B3, New York, NY 10032 USA; 2 Icahn School of Medicine at Mt Sinai, Queens Hospital Center Department of Emergency Medicine, 82-68 164th Street, Suite 1B-02, Queens, NY 11432 USA; 3 Center for Refugee and Disaster Response, Johns Hopkins University Bloomberg School of Public Health, 615 N Wolfe Street, Baltimore, MD 21205 USA; 4 World Health Organisation, Avenue Appia 20, 1211 Geneva 27, Switzerland; 5 Harvard Humanitarian Initiative, 14 Story St, Cambridge, MA 02138 USA; 6 Brigham & Women’s Hospital Department of Emergency Medicine, 75 Francis Street, Neville House 2nd Floor, Boston, MA 02115 USA

**Keywords:** Health spending, Refugees, Mortality, Health information system

## Abstract

**Background:**

Numerous simultaneous complex humanitarian emergencies strain the ability of local governments and the international community to respond, underscoring the importance of cost-effective use of limited resources. At the end of 2011, 42.5 million people were forcibly displaced, including 10.4 million refugees under the mandate of the United Nations High Commissioner for Refugees (UNHCR). UNHCR spent US$1.65 billion on refugee programs in 2011. We analyze the impact of aggregate-level UNHCR spending on mortality of refugee populations.

**Methods:**

Using 2011 budget data, we calculated purchasing power parity adjusted spending, disaggregated by population planning groups (PPGs) and UNHCR Results Framework objectives. Monthly mortality reported to UNHCR’s Health Information System from 2011 to 2012 was used to calculate crude (CMR) and under-5 (U5MR) mortality rates, and expressed as ratios to country of asylum mortality. Log-linear regressions were performed to assess correlation between spending and mortality.

**Results:**

Mortality data for 70 refugee sites representing 1.6 million refugees in 17 countries were matched to 20 PPGs. Median 2011 spending was $623.27 per person (constant 2011 US$). Median CMR was 2.4 deaths per 1,000 persons per year; median U5MR was 18.1 under-5 deaths per 1,000 live births per year. CMR was negatively correlated with total spending (*p =* 0.027), and spending for fair protection processes and documentation (*p =* 0.005), external relations (*p =* 0.034), logistics and operations support (*p =* 0.007), and for healthcare (*p =* 0.046). U5MR ratio was negatively correlated with total spending (*p =* 0.015), and spending for favorable protection environment (*p =* 0.024), fair protection processes and documentation (*p =* 0.003), basic needs and essential services (*p =* 0.027), and within basic needs, for healthcare services (*p =* 0.007).

**Conclusion:**

Increased UNHCR spending on refugee populations is correlated with lower mortality, likely reflecting unique refugee vulnerabilities and dependence on aid. Future analyses using more granular data can further elucidate the health impact of humanitarian sector spending, thereby guiding policy choices.

**Electronic supplementary material:**

The online version of this article (doi:10.1186/s13031-016-0095-4) contains supplementary material, which is available to authorized users.

## Background

At the end of 2011, an estimated 42.5 million people were considered forcibly displaced, including 15.2 million refugees. Of these refugees, 10.4 million fell under the mandate of the United Nations High Commissioner for Refugees (UNHCR) [1]. Over US$2.1 billion was spent by UNHCR in 2011 to protect and seek permanent solutions for refugees and other persons of concern, of which US$1.65 billion was spent on refugee programs [2]. To strengthen accountability for its programs and spending, UNHCR recently developed a standardized Results Framework that describes the results targeted by UNHCR organized into nine rights groups [3]. This Results Framework and the organization of the operating budget by rights groups allows for an assessment of the impact of UNHCR spending on its goals. To date, however, the impact of global UNHCR spending on health outcomes has not been analyzed.

Focusing on UNHCR’s spending effects in terms of the health of refugee populations is of particular interest for many reasons. First, protection of refugee populations including their right to health represents a core priority of the organization and of the greater humanitarian community. UNHCR budgeting and spending reflects this priority—the largest proportion of UNHCR spending within the Results Framework is devoted to the “Basic Needs and Essential Services” rights group, which includes programs ranging from primary healthcare services to water and sanitation to education, and comprises approximately 34 % of the total spending in 2012 [4]. Second, health outcomes reflect direct as well as indirect interventions. Efforts to ensure the legal rights of refugees or simply registering refugees in a host country, for example, may have direct and indirect effects on improving access to vaccinations, food, education, or shelter, thereby ultimately affecting the health and well-being of refugee populations. Third, health outcomes are generally well-defined, objective, and extensively studied indicators of population well-being.

Prior studies of the impact of population-level spending on health outcomes provide a background within which this analysis can be understood. The evidence supporting the impact of health spending on non-conflict affected large populations is mixed. Analyses of several specific health interventions and vertical programs suggest that these interventions can have a significant cost-effective impact on reducing morbidity and mortality [5–7]. At a global level, however, cross-national regression analyses of public health-sector spending and health outcomes indicate that the effect is small or non-existent, and is associated with a high cost per death averted [8]. Yet a few cross-national analyses focusing on certain subsets of countries, such as low-income countries [9] or focus countries of the President’s Emergency Plan for AIDS Relief, [10] suggest a potential correlation between public health spending or health-sector foreign aid and reduced mortality. Differences between health outcomes at the national or population level are accounted for primarily by socioeconomic factors such as wealth, education, and geography [8].

Refugees, internally displaced persons, and other persons of concern falling under UNHCR’s mandate represent a unique type of population. Socioeconomic factors are disturbed by loss of property and sources of livelihood, dependence on humanitarian aid, and other effects of forced migration [11]. In addition, populations living in camps, sites, and settlements—henceforth called sites for this paper—are particularly dependent and sensitive to the health infrastructure established for them by governments, UN agencies such as UNHCR, and local and international non-governmental organizations (NGOs).

Existing studies of risk factors associated with refugee health outcomes have focused on public health variables such as access to water and latrines, distance to health facilities, and health service utilization [12, 13]. While such features of the public health environment are undoubtedly important, they in turn participate within a broader refugee site economy that is distinct from mainstream economies due to barriers to refugee employment, difficulty participating in markets and trade, and lack of representation in governance and policy-making [11, 14]. As a result, refugee health is particularly sensitive to foreign aid in the form of food and non-food assistance, water and sanitation investments, healthcare services, and other forms of humanitarian assistance, as well as the policy decisions governing this aid spending. Yet, we do not know if such population-level spending on refugees matters in terms of their health outcomes.

To assess the impact of spending on refugee population health outcomes, we analyze budget data from UNHCR’s results-based management software, *Focus*, and health outcome data drawn from UNHCR’s Health Information System, or HIS (now part of the Twine program) [15]. We hypothesize that total spending on refugee site populations and more specifically spending on basic needs and essential services is positively correlated with improved health outcomes as measured by crude and under-5 mortality, thus reflecting the greater dependency of refugee populations on the provision of services through humanitarian aid, and differing from the findings of the aforementioned cross-national studies.

## Methods

### Health information system

Health outcome data are derived from HIS, which is a standardized public health surveillance tool used by UNHCR to inform policy decisions and health program management [15]. As of year-end 2012, data from 153 refugee sites in 25 countries representing a population of 2.6 million persons of concern were reported into HIS. Monthly data from HIS from January 1, 2011 through December 31, 2012 were exported into Microsoft Excel, which was used for data exploring, cleaning, and aggregation. Data fields exported from HIS included site name, country of asylum, month and year of reporting, site population, number of live births, crude mortality rate (per 1,000 persons per month), and under-5 mortality rate (per 1,000 children under-5 years per month).

Of the 153 refugee sites with data reported to HIS from January 2011 through December 2012, sites were included if greater than 12 out of 24 months of data were reported. This resulted in 91 included sites. Data for each of these 91 sites were assessed individually for missing or inconsistent data, resulting in the exclusion of 3 sites due to missing mortality figures, and 12 sites due to logical inconsistencies in population reporting (for example, number of live births greater than female population). Sites were then matched to Population Planning Groups (PPGs). A PPG is an internal UNHCR population designation representing a group of persons of concern for which spending and programming decisions are made. PPG definitions range from individual refugee sites (“refugees and asylum seekers of various nationalities in Kakuma camp”), to ethnic and/or geographic descriptions representing several sites (“Sudanese refugees in the East of Chad”). Of the sites thus far not excluded, 71 sites were matched to 21 PPGs; one site contained refugees from two different PPGs, and four sites could not be matched to a PPG, and were thus excluded. PPGs were often made up of multiple refugee sites, so to ensure that the sites reported upon in HIS were representative of their matched PPG, only PPGs for which the midpoint population captured in HIS represented at least one-quarter of the total PPG population were included in the final analysis. This criterion excluded one PPG (“Central African refugees in the East and Adamaoua, Cameroon”), for which the sites in HIS represented only 10 % of the total PPG population; for all of the other 20 included PPGs, matched HIS data represented 31 to 149 % of their respective PPG populations (median 90 %; some HIS populations were greater than PPG populations due to influx of refugees).

Health data reported in HIS is collected through prospective surveillance in health facilities. Each death is classified according to direct and indirect cause(s) and recorded in a central mortality register. These register records are then aggregated to weekly totals and submitted by each health partner in a routine weekly report with other HIS data. This method of mortality data collection is subject to a number of biases, the most significant being a tendency towards under-reporting of community-based deaths that are not notified to a health facility. There are many cultural, social, and economic reasons why families may wish to not report a death to camp authorities. To improve reporting, HIS standards require that a central mortality register should be maintained in each site and triangulated with other mortality sources (such as shroud distribution records and graveyard records) to ensure all deaths are recorded. Also, some camp authorities only permit burial of bodies that have been issued a death certificate or death notification by the local Ministry of Health or similar governmental health partner. To incentivize family reporting of deaths, families that declare deaths to camp authorities are provided with burial materials and shrouds to assist with the burial. In some sites, UNHCR agrees to delay the removal of ration cards from deceased persons, so families can continue to benefit from ration distributions for 3 to 6 months after death.

For each site included in HIS, the monthly crude mortality rate and monthly population was used to calculate an annualized crude mortality rate based on the midpoint population and taking into account the number of months out of 24 reported in HIS. These site-level annualized crude mortality rates (CMR) were then re-aggregated by PPG, thus resulting in a crude mortality rate for the PPG over the 2-year study period, and reported as deaths per 1,000 persons per year. A similar process was used to calculate the annualized under-5 mortality rate (U5MR) for each site, except that the total number of live births was used in the denominator in place of the under-5 population. Site-level under-5 mortality rates were re-aggregated by PPG, and reported as under-5 deaths per 1,000 live births per year.

### UNHCR budget

The UNHCR budget process involves several stages, starting with development of strategic plans based on a comprehensive assessment of needs in all UNHCR operations beginning 1 year before the budget year, approval of plans and resource requirements after corporate review, progressing to preparation of more detailed plans and budgets in the months before the beginning of the budget year [2]. At the start of the budget year, operations are issued with budget authorization (spending authority) based on the projected income of global resources to the operations. For operations with emergency needs, additional funding may be made available in installments at later stages in the implementation year subject to availability. The budget data used in this paper are drawn from UNHCR’s *Focus* software and reflects the “detailed plan” budget stage, corresponding to the final phased operational spending plan in the budget process, and thus closely approximates actual spending. For example, the total amount of money spent as reported in the UNHCR Global Report 2011 [2] was 95.9 % of the total amount included in Focus at the “detailed plan” budget level. In order to address reverse causation bias in which mortality might influence budgeted spending levels, we used budget and mortality data staggered by time. Data from UNHCR’s 2011 budget year are used, reflecting needs assessments, strategic planning, and policy decisions from the year 2010 and earlier. Thus, we make the assumption that budget allocation decisions in 2010 and earlier are not impacted by health outcomes data observed from January 1, 2011 to December 31, 2012.

Budget data in UNHCR’s *Focus* results-based management software were disaggregated by PPG and by objective within the Results Framework (see Table 1), then exported into Excel, which was used to calculate per capita spending, by PPG and by objective, based on population figures from PPG planning documents. Per capita spending figures were then corrected for purchasing power parity (PPP) based on indices from the Penn World Table 8.1, [16] and expressed in constant 2011 US dollars.

**Table 1:** Objectives from UNHCR Results Framework used to disaggregate budget

• Favorable protection environment	
• Fair protection processes and documentation	
• Security from violence and exploitation	
• Community participation and self-management	
• Durable solutions	
• External relations	
• Logistics and operations support	
• Headquarters and regional support (excluded because no spending was allocated to this budget category in the 20 included PPGs)	
• Basic needs and essential services—further broken down into the following: - Water & sanitation (“supply of potable water increased or maintained” and “population lives in satisfactory sanitary conditions”) - Education (“population has optimal access to education”) - Shelter/infrastructure (“shelter and infrastructure improved”) - Non-food items (“population has sufficient basic domestic and hygiene items”) - Food security & nutrition (“food security improved” and “nutritional well-being improved”) - Healthcare services (“health of the population improves or remains stable” and “risk of HIV/AIDS reduced and quality of response improved”)	

### Statistical analysis

CMR and U5MR data from the HIS database and UNHCR budget data from Focus were analyzed in STATA version 13.1 (StataCorp, College Station, Texas, USA). Log-linear regressions were performed using mortality as the outcome variable, and UNHCR budget as the independent variable.

For the mortality outcome, the CMR was expressed as a ratio of refugee CMR to country of asylum CMR using national CMR figures from the World Bank [17]. This ratio was used in order to derive an outcome expressing refugee health relative to baseline health as approximated by the host population CMR, and reflects the Sphere standard of comparing mortality indicators to baseline rates prior to the disaster [18]. Similarly, U5MR was also expressed as a ratio of refugee U5MR to country of asylum U5MR. Country of asylum mortality, rather than country of origin mortality, was used as the basis of comparison in these mortality ratios since many PPGs consisted of refugees from several different countries of origin.

The budget variable was expressed as PPP-adjusted per capita total budget and per capita objective-specific budget based on objectives within the Results Framework, and log transformed. Separate log-linear regressions were performed using each mortality outcome and each budget category, using regression equations of the following basic form:

$$ ln\left(\frac{refugee\; mortality}{country\; of\; asylum\; mortality}\right)={\beta}_0+{\beta}_1\cdot ln(budget)+\varepsilon . $$

Thus, each regression is a cross-sectional analysis of between-PPG differences which evaluates if the level of refugee mortality relative to country of asylum mortality is associated with the level of per capita budgeted spending in the study year.

In addition to separate regressions for each budget category, a secondary analysis was performed using seemingly unrelated regression to estimate a system of equations simultaneously, and is reported in further detail in the Additional file 1.

Mortality figures were entered into the regression analysis as point estimates, and the resulting regression coefficients and *p*-values were used to describe the association between mortality and budgeted spending. In order to propagate the uncertainty of the mortality figures through the regression analysis, a Monte Carlo simulation method was used. The distribution of each mortality observation was sampled 1,000 times. Each regression analysis was then performed 1,000 times using the sample of mortality figures, and the distribution of the resulting 1,000 regression coefficients was used to calculate 95 % confidence intervals for the regression coefficients.

## Results

The health outcomes data from HIS were assembled from 70 refugee sites representing 1.6 million refugees living in 17 different host countries in Africa, South and Southeast Asia, and the Middle East and North Africa. The CMR and U5MR for the 20 PPGs for 2011–2012 are presented in Table 2. The CMR of the PPGs ranged from 0.5 to 4.9 deaths per 1,000 persons per year, with a median CMR of 2.4. The U5MR of the PPGs ranged from 2.5 to 40.6 under-5 deaths per 1,000 live births per year, with a median U5MR of 18.1. In general, the CMRs and U5MRs calculated from the HIS data tended to be lower than country of asylum CMRs and U5MRs, with mortality rate ratios ranging from 0.061 to 0.551 for crude mortality, and 0.035 to 0.723 for under-5 mortality.

**Table 2:** Crude and under-5 mortality rates by population planning groups (PPG) for 1.6 million refugees living in 17 different host countries, 2011–2012

Country of asylum	PPG description	CMR^a^ (95 % CI)	U5MR^b^ (95 % CI)
Burundi	Congolese refugees and asylum seekers	2.6 (2.0 to 3.3)	40.6 (27.1 to 54.0)
Bangladesh	Refugees from Northern Rakhine State, Myanmar	3.0 (2.4 to 3.6)	8.8 (4.6 to 13.1)
Central African Republic	Refugees and asylum seekers in rural areas	4.9 (3.0 to 6.8)	31.1 (9.9 to 52.4)
Djibouti	Refugees in Djibouti	1.0 (0.5 to 1.4)	8.9 (1.4 to 16.4)
Ethiopia	Eritrean refugees and asylum seekers	1.1 (0.7 to 1.6)	9.4 (3.1 to 15.7)
Ethiopia	Somali refugees and asylum seekers	1.8 (1.5 to 2.1)	21.8 (17.6 to 26.1)
Ethiopia	Mainly Sudanese refugees in Western Ethiopia	0.5 (0.2 to 0.8)	2.5 (0 to 5.1)
Kenya	Refugees and asylum seekers of various nationalities in Kakuma camp	2.2 (1.9 to 2.5)	17.9 (14.3 to 21.5)
Kenya	Mainly Somali refugees and asylum seekers	2.8 (2.7 to 3.0)	27.7 (25.4 to 30.0)
Namibia	Refugees and asylum seekers in Osire camp and Windhoek	2.0 (1.0 to 3.1)	27.2 (4.3 to 50.2)
Nepal	Refugees and asylum seekers from Bhutan	3.8 (3.3 to 4.2)	10.2 (5.3 to 15.2)
Rwanda	Refugees in camps	2.1 (1.7 to 2.5)	14.8 (9.5 to 20.0)
Sudan	Refugees and asylum seekers in the East	3.8 (3.3 to 4.2)	21.3 (16.8 to 25.7)
Chad	Refugees from the Central African Republic	3.2 (2.8 to 3.7)	34.0 (27.3 to 40.8)
Chad	Sudanese refugees in the East	2.7 (2.5 to 2.9)	18.3 (16.4 to 20.2)
Thailand	Myanmar refugees in border camps	3.2 (2.9 to 3.5)	9.9 (7.7 to 12.1)
Tanzania	Refugees and asylum seekers in camps	3.4 (3.1 to 3.8)	20.0 (16.9 to 23.2)
Uganda	Congolese, Rwandan, Somali, Burundian and other refugees	1.2 (0.9 to 1.4)	9.5 (6.9 to 12.1)
Yemen	Refugees	2.1 (1.8 to 2.5)	17.0 (12.6 to 21.4)
Zambia	Refugees of various origins in camps	1.2 (0.8 to 1.6)	18.6 (10.8 to 26.4)

^a^ Deaths per 1,000 persons per year

^b^ Under-5 child deaths per 1,000 live births per year

Total per capita UNHCR budgeted spending, budgets for basic needs and essential services, and the health-sector component of basic needs spending are listed in Table 3. The PPP-adjusted UNHCR budgeted spending per capita for 2011 in the 20 included PPGs ranged from US$231.33 to US$2055.63, with a median budget of $623.27 per person (in 2011 US$). Spending allocated for basic needs and essential services was generally a significant portion of the budget for each PPG, ranging from 19 to 68 % of the total, and was the largest category of budgeted spending for 16 (80 %) of the 20 PPGs.

**Table 3:** UNHCR 2011 budgeted spending per capita (PPP-adjusted 2011 US$): total, basic needs, and healthcare by population planning groups (PPG) for 1.6 million refugees living in 17 different host countries

Country of asylum	PPG description	Total	Basic needs	Healthcare
Burundi	Congolese refugees and asylum seekers	$980.13	$366.91	$127.39
Bangladesh	Refugees from Northern Rakhine State, Myanmar	$686.63	$398.17	$85.45
Central African Republic	Refugees and asylum seekers in rural areas	$614.81	$337.68	$121.29
Djibouti	Refugees in Djibouti	$1,829.75	$1,027.15	$139.26
Ethiopia	Eritrean refugees and asylum seekers	$939.97	$489.94	$139.36
Ethiopia	Somali refugees and asylum seekers	$2,047.61	$1,345.96	$173.65
Ethiopia	Mainly Sudanese refugees in Western Ethiopia	$2,055.63	$1,022.57	$222.67
Kenya	Refugees and asylum seekers of various nationalities in Kakuma camp	$538.81	$241.55	$51.34
Kenya	Majority Somali refugees and asylum seekers	$867.19	$586.74	$78.95
Namibia	Refugees and asylum seekers in Osire camp and Windhoek	$463.01	$226.85	$47.27
Nepal	Refugees and asylum seekers from Bhutan	$399.54	$196.21	$53.70
Rwanda	Refugees in camps	$483.44	$228.37	$51.09
Sudan	Refugees and asylum seekers in the East	$631.72	$251.23	$64.99
Chad	Refugees from the Central African Republic	$739.35	$178.26	$55.93
Chad	Sudanese refugees in the East	$735.61	$275.63	$78.36
Thailand	Myanmar refugees in border camps	$231.33	$49.96	$7.28
Tanzania	Refugees and asylum seekers in camps	$444.56	$83.46	$12.39
Uganda	Congolese, Rwandan, Somali, Burundian and other refugees	$460.46	$211.30	$65.94
Yemen	Refugees	$271.01	$127.54	$23.17
Zambia	Refugees of various origins in camps	$316.11	$82.70	$25.91

Scatter plots of mortality vs. budgeted spending for each of the spending categories are reported in the Additional file 1.

The results of regression analyses correlating health outcomes with various categories of UNHCR budgeted spending are presented in Table 4. CMR was found to have a statistically significant correlation with total budgeted spending (*p =* 0.027), and budgeted spending for fair protection processes and documentation (*p =* 0.005), external relations (*p =* 0.034), and logistics and operations support (*p =* 0.007). For each of these correlations, the estimated regression coefficients were negative values, indicating that more budgeted spending was associated with lower mortality. Within the basic needs budget category, CMR was significantly correlated with budgeted spending for healthcare services (*p =* 0.046), also with a negative estimated regression coefficient. Budgeted spending for other aspects of basic needs and services were not correlated with decreased mortality, though the correlation with budgeted spending for water and sanitation trended towards significance (*p =* 0.057).

**Table 4:** Regression results estimating correlation between budgeted spending and health outcomes by population planning groups (PPG) for 1.6 million refugees living in 17 different host countries, 2011–2012

	Health outcomes			
Budget category^a^ (n) ^b^	Crude mortality^c^		Under-5 mortality^d^	
	b_1_ (95 % CI)	*p*-value	b_1_ (95 % CI)	*p*-value
Total (*n =* 20)	−0.469 (−0.629 to−0.332)	0.027^e^	−0.580 (−0.902 to−0.284)	0.015^e^
Favorable protection environment (*n =* 20)	−0.342 (−0.528 to−0.172)	0.124	−0.553 (−0.925 to−0.188)	0.024^e^
Fair protection processes and documentation (*n =* 20)	−0.515 (−0.692 to −0.360)	0.005^e^	−0.613 (−0.966 to−0.279)	0.003^e^
Security from violence and exploitation (*n =* 20)	−0.180 (−0.307 to−0.064)	0.378	−0.237 (−0.486 to 0.009)	0.306
Community participation and self-management (*n =* 20)	−0.087 (−0.166 to−0.016)	0.651	−0.183 (−0.331 to−0.048)	0.400
Durable solutions (*n =* 20)	−0.271 (−0.384 to−0.169)	0.278	−0.251 (−0.455 to−0.064)	0.382
External relations (*n =* 17)	−0.462 (−0.640 to−0.313)	0.034^e^	−0.533 (−0.889 to−0.213)	0.051
Logistics and operations support (*n =* 20)	−0.375 (−0.465 to−0.298)	0.007^e^	−0.313 (−0.496 to−0.137)	0.060
Basic needs and essential services (*n =* 20)	−0.267 (−0.369 to−0.181)	0.088	−0.385 (−0.588 to−0.207)	0.027^e^
- Water & sanitation (*n =* 19)	−0.238 (−0.325 to−0.163)	0.057	−0.257 (−0.433 to−0.097)	0.056
- Education (*n =* 20)	−0.226 (−0.318 to−0.150)	0.155	−0.212 (−0.387 to−0.060)	0.248
- Shelter/infrastructure (*n =* 20)	−0.101 (−0.160 to−0.048)	0.372	−0.225 (−0.338 to−0.117)	0.073
- Non-food items (*n =* 14)	−0.178 (−0.264 to−0.102)	0.128	−0.195 (−0.363 to−0.036)	0.146
- Food security & nutrition (*n =* 20)	0.041 (−0.050 to 0.132)	0.806	−0.128 (−0.297 to 0.050)	0.509
- Healthcare services (*n =* 20)	−0.305 (−0.399 to−0.227)	0.046^e^	−0.449 (−0.636 to−0.289)	0.007^e^

^a^ Natural log of budgeted spending per capita, PPP-adjusted 2011 US$

^b^ *n =* number of included PPGs; dropped if spending in PPG for that budget category was $0

^c^ Natural log of ratio of PPG crude mortality rate to country of asylum crude mortality rate

^d^ Natural log of ratio of PPG under-5 mortality rate to country of asylum under-5 mortality rate

^e^ Statistically significant at α = 0.05 level

Similar results were found for regression analyses using U5MR as the outcome measure. U5MR was correlated with total budgeted spending (*p =* 0.015), and budgeted spending for favorable protection environment (*p =* 0.024), fair protection processes and documentation (*p =* 0.003), and basic needs and essential services (*p =* 0.027), and within the basic needs category, for healthcare services spending (*p =* 0.007). As with CMR, the regression coefficients describing the correlation between budgeted spending categories and U5MR were also all negative values, suggesting that for the included PPGs, higher levels of budgeted spending were associated with lower under-5 mortality.

## Discussion

There are currently numerous large scale and complex simultaneous humanitarian emergencies such as in Syria, Iraq, South Sudan, and Central African Republic. These have strained the host governments’ and international community’s ability to respond adequately, both in terms of personnel, infrastructure and services, and funding. Never before has it been more important to use precious and limited funds in a cost-effective manner to respond to humanitarian crises. Increasingly, large bilateral and multilateral donors are examining ‘value for money’ [19]. This study shows that for a refugee population represented by 20 PPGs including 1.6 million refugees living in 70 refugee sites in 17 different host countries in Africa, South and Southeast Asia, and the Middle East and North Africa, increased system-wide funding for refugee services is positively associated with improved health outcomes.

The results of the regression analyses suggest that total UNHCR spending on protection and assistance programs is positively correlated with a reduction in CMR and U5MR, meaning that higher levels of budgeted spending tended to occur together with better relative health outcomes more frequently than would be expected by chance. Furthermore, increased spending within specific budget categories was found to positively correlate with reduced mortality rates; budgeting for fair protection processes and documentation, and for healthcare services were correlated with both CMR and U5MR; budgeting for external relations, and for logistics and operations support was correlated with CMR; and budgeting for favorable protection environment and for basic needs and essential services was correlated with U5MR. Although these results should not be taken as a definitive recommendation to increase spending in these specific areas, the implications and limitations of these findings raise important considerations for funding programs in humanitarian response.

While spending on basic needs and essential services such as water and sanitation or healthcare would be expected to correlate with health outcomes, the connection between other budget categories and mortality is indirect. Within UNHCR’s Results Framework, fair protection processes and documentation includes activities targeted at improving identification and registration of persons of concern [20]. Since the right to access services and assistance is often tied to documentation of status, effective status determination may improve the ability for crucial assistance such as food, shelter, medicines, and education to reach its intended recipients. Also, effective and complete registration will in turn lead to accurate population planning figures, thus avoiding the problem of inadequate aid allocated because of an undercount of the number of refugees. Spending in the logistics and operations support budget category, which we also found correlated with CMR, should improve the efficiency of service delivery, leading to more effective programs in shelter, water and sanitation, healthcare, and other health-related domains thereby reducing mortality.

Unlike assessments of the impact of individual vertical programs, the analysis presented in this paper evaluates budgeted spending for UNHCR activities at a system-wide, aggregate level. Thus, the findings take into account the potential for loss of effectiveness resulting from “real-world” inefficiencies that occur at each level of the causal pathway between spending and health outcomes. These inefficiencies might arise from poor policy decisions or funding choices, ineffective creation of intermediate outputs or service delivery, the crowding-out of other service providers, or lack of efficacy of the interventions themselves [21]. Such aggregate level analysis more closely resembles the numerous studies of the cross-national impact of aid or public health spending, for which these inefficiencies have been used to explain the empirical lack of correlation between foreign aid and economic growth, or between public spending on health and health outcomes [8, 21, 22].

The results suggest that even when assessed at an aggregate level and including the effect of these real-world inefficiencies, higher levels of UNHCR budgeted spending has an impact on improved health outcomes. This differs from the results of cross-national analyses of the impact of spending on health involving all countries [8, 22], but mirrors analyses limited to more vulnerable, disadvantaged countries or populations [9, 10]. Our finding that refugee program spending has an impact on health outcomes thus likely reflects the unique vulnerability of refugee populations. Dependence on assistance and services provided by humanitarian organizations to refugees, as well as barriers preventing refugees from working or accessing services outside of a refugee site [11, 14], cause refugee populations to be more sensitive to changes in the level of assistance provided by host governments and humanitarian organizations. In addition, the relationship between UNHCR and partner NGOs is vastly different from a typical competitive market; rather than crowding-out other service providers, money spent by UNHCR for services contracted from another organization may in fact “crowd-in” additional services and spending as the partner organization brings its own donor funds to supplement its programming. The geography and organization of a refugee site may also improve the population access and efficacy of certain services such as vaccination, nutrition and food assistance, and access to healthcare, as was observed in a prior study comparing outpatient healthcare service utilization between refugees and the host population [13].

These results should be interpreted within the context of the protracted or chronic phase of a humanitarian situation. This is because the study sample was biased towards more stable refugee sites, reflecting the time needed for setup and implementation of HIS; the exclusion of sites with missing or inconsistent data likely added to this bias. Though this limits the ability to apply these results to all refugee situations, we theorized that unstable refugee situations would have uncharacteristic spending and mortality levels that are more reflective of immediate threats and shocks to the population rather than any stable relationship between spending and health outcomes.

The overall CMRs and U5MRs calculated from the HIS data were low, which was especially apparent when compared to the corresponding mortality rates of the country of asylum. While it has been reported previously that health outcomes among refugees are often better than the health outcomes of their hosts [23, 24], especially in the protracted or chronic phase of a refugee situation, this factor alone does not likely account for the size of the difference between the calculated PPG mortality rates and the country of asylum mortality rates. Another potential reason for the low reported mortality rates is the tendency for surveillance systems such as HIS to underestimate the true mortality rate, as some deaths will not be identified by the formal reporting system [25]. Furthermore, refugee site population censuses tend to be overestimated for a variety of reasons including double counting of refugees and the inclusion of host populations [25, 26]. Thus, the denominator for crude mortality rate calculations is often inflated, thereby further decreasing the calculated rate; this is not the case for under-5 mortality, as live births are used for calculating the denominator in this study, and live births tend to be reliably registered since they translate to increases in food rations.

With assumptions of PPG population size, refugee mortality rates, and country of asylum mortality rates, the estimated regression function can be used to express the health impact of UNHCR budgeted spending in more typical cost-effectiveness terms, such as cost per death averted. For example, if median figures for PPG population and mortality are used, the regression results suggest that increasing spending on healthcare services by an average of US$44,274 (95 %CI: US$31,456 – US$57,091) per year would have resulted in one fewer death. This figure is high when compared to the estimated cost per death averted for the most cost-effective interventions in areas with high disease burden, ranging from a few hundred to a few thousand US dollars [7]. As explained previously, however, the analysis presented in this paper is performed at an aggregate level, and includes the costs of inefficiencies in translating spending into outputs and impact. When compared to the cost per death averted for public spending on health at the cross-national level, estimated at US$50,000 to US$100,000 per year in developing countries [8], this figure falls just below the expected range. The cost per death averted estimated by this analysis, however, may be inflated by the lower than expected mortality rates derived from the HIS data, which may be underreported for the reasons discussed in the previous paragraph. Though this analysis was not designed to estimate cost-effectiveness of spending on refugee healthcare services, the finding that the extrapolated cost per death averted is below the estimated range is nevertheless consistent with the notion of refugee populations being uniquely vulnerable and sensitive to aid spending, and warrants further study.

This analysis was subject to several limitations. First, budget data, rather than actual expense data, were used to reflect the costs to UNHCR. While the phased budget process used by UNHCR ensures that the budget at the “detailed plan” stage closely approximates actual spending (and in fact differs in aggregate by less than 5 %), expenditure data would have been preferable. Furthermore, only UNHCR budgeted spending is included in this analysis; spending from other sources such as NGOs, the host government, private remittances, or local trade were not included. It is unclear if UNHCR spending has a crowding-out or crowding-in effect on spending from partner organizations. At the cross-national level, foreign development assistance for health has been previously reported to have varying effects on increasing or decreasing domestic government health spending depending on whether the assistance was provided to the government or the NGO sector [27]. Thus, the results can only be interpreted in terms of UNHCR spending, and not the overall health impact of humanitarian sector spending in these 70 refugee sites.

Second, the cross-sectional design of the regression analysis limits the findings to correlation; a causal relationship between spending and health outcomes cannot be determined. Reverse causation is another possibility in which health outcomes determine budget levels, since health data from HIS is one of many potential factors considered by UNHCR in the budgeting process. By using budget data for 2011, reflecting a needs assessment and priority-setting process from 2009 to 2010, we attempted to address reverse causation by assuming based on temporal ordering that 2011–2012 mortality did not affect spending levels budgeted before 2011. This assumption potentially breaks down, however, if past mortality rates are correlated to future mortality rates. To test if past and future mortality rates are correlated, we calculated Pearson’s correlation coefficient for CMR and U5MR in 2011 vs. 2012 across all HIS sites included in the analysis. For CMR, the correlation coefficient is 0.368 (*p =* 0.001), which is significantly different from the null hypothesis of zero correlation at the α = 0.05 level, but would be considered a “weak” correlation by Evans’ classification [28]. For U5MR, the correlation coefficient is 0.146 (*p =* 0.218), which is not statistically significant, and would be classified a “very weak” correlation.

Third, a weakness in the mortality figures calculated from the HIS data is the lack of age and gender standardization that would especially affect CMR. Variation in the age and gender distributions between site or PPG populations can alter death rates in a manner that does not reflect actual population health status. By using ratios of refugee mortality to country of asylum mortality, we partially correct for being unable to standardize the mortality rates. This assumes that the age and gender distributions of refugee populations are similar to the country of asylum population, or at least dissimilar to an equal degree, though this might not actually be the case.

Fourth, because the regression model used log transformations of the budget variable, several PPGs were dropped from certain objective-specific analyses when the budgeted spending for that objective was $0. This occurred in regressions involving budgeted spending for external relations, water and sanitation, and non-food items, and thus the results for these specific budget categories do not reflect the entire dataset.

Fifth, the granularity of the budget data in *Focus* was limited to the PPG level. As a result, health outcome data had to be derived by aggregating HIS data from the site level to the PPG level, so the analysis does not take into account differences between sites within the same PPG. Additionally, analysis at the PPG level limited the number of observations in the regression, despite the HIS data representing a relatively large number of refugee sites. Ideally, multiple variable regression analyses could adjust for known correlates of mortality such as education level, access to water, HIV prevalence, and geography, thus allowing for a more robust model. Such multiple variable models could not be used with the small number of available observations (20 PPGs), however, as the resulting few degrees of freedom would increase risk of an over-fitted model. Furthermore, data on these potential covariates at the PPG level for all of the included refugee sites was not available to the authors. Also, the budget data from *Focus* used in this analysis was limited to a single year because UNHCR PPG definitions shift from year to year, making budget allocations incomparable between years. If several comparable years of budget data were available, this would allow for a greater sample size using the cross-sectional approach described in this paper for analyzing between-PPG differences in mortality and spending; alternatively, multiple years of budget data could also allow for panel data analyses looking at within-PPG differences over time. Though the results-based organization of the budget in *Focus* makes a health impact analysis of UNHCR spending possible, future studies may benefit from budget or expenditure data disaggregated by sites, and time series data using stable PPG definitions from one budget year to the next.

## Conclusions

Through the results-based reporting of UNHCR budgeted spending and health outcomes data available in the HIS database, we analyzed the health impact of UNHCR spending on refugee programs at an aggregate level. The results show that increased UNHCR budgeted spending correlates with reduced mortality, including total spending, spending on fair protection processes and documentation, and healthcare spending for both CMR and U5MR. Furthermore, spending on external relations and logistics and operations support were correlated with a reduction in CMR, and spending on favorable protection environment and basic needs and essential services were correlated with a reduction in U5MR. The calculated cost per death averted in terms of UNHCR spending on healthcare falls slightly below the range estimated by other cross-national analyses of the impact of aggregate-level public spending on health.

Future studies using more granular data can further elucidate the health impact of spending in the humanitarian sector, and potentially guide international community policy decisions and intervention prioritization. Studies of the health impact of programmatic spending such as this one are rare in the world of humanitarian response. However, in the current situation of multiple and simultaneous large scale crises with consequent limited human and financial resources, such studies are needed to ensure that affected populations receive the most cost-effective interventions possible including healthcare, and suffer less mortality.

## Additional file

Additional file 1: Supplemental figures and analysis. (DOCX 214 kb)

## Abbreviations

CMR: Crude mortality rate
HIS: Health information system
NGO: Non-governmental organization
PPG: Population planning group
PPP: Purchasing power parity
U5MR: Under-5 mortality rate
UNHCR: United Nations high commissioner for refugees
