# Supplementary material for: Does spending on refugees make a difference? A cross-sectional study of the association between refugee program spending and health outcomes in 70 sites in 17 countries
Source: Confl Health. 2016 Dec 7;10:28. doi: 10.1186/s13031-016-0095-4 (PMC5477838; doi:10.1186/s13031-016-0095-4)
Supplement: Additional file 1: — Supplemental figures and analysis. (DOCX 214 kb) [file 13031_2016_95_MOESM1_ESM.docx]

**Supplemental figures and analysis for “Does spending on refugees make a difference? A cross-sectional study of the association between refugee program spending and health outcomes in 70 sites in 17 countries”**

**Supplemental scatter plots**

The basic relationship analyzed in this paper is the level of mortality in refugee sites associated with various levels of per capita spending for refugee programs. Scatter plots depicting these relationships are provided below. In each plot, the y-axis depicts the ratio of mortality observed in the refugee site to the mortality rate of the country of asylum. Per capita spending by UNHCR in PPP-adjusted 2011 US$ is depicted on the x-axis. Multiple plots are shown for various budget categories sub-divided by the objectives used by UNHCR in its results framework (see **Table 1** in main paper). Within the analysis for each budget category, two plots are depicted, one using crude mortality rates in the refugee to country of asylum mortality ratio, and the second using under-5 child mortality.

***Figures 1a and 1b: Mortality ratio vs total spending per capita***

***Figures 2a and 2b: Mortality ratio vs spending per capita on favorable protection environment***

***Figures 3a and 3b: Mortality ratio vs spending per capita on fair protection processes and documentation***

***Figures 4a and 4b: Mortality ratio vs spending per capita on security from violence and exploitation***

***Figures 5a and 5b: Mortality ratio vs spending per capita on community participation and self-management***

***Figures 6a and 6b: Mortality ratio vs spending per capita on durable solutions***

***Figures 7a and 7b: Mortality ratio vs spending per capita on external relations***

***Figures 8a and 8b: Mortality ratio vs spending per capita on logistics and operations support***

***Figures 9a and 9b: Mortality ratio vs spending per capita on basic needs and essential services***

For Figures 10 to 15, the spending on the x-axis refers to spending budgeted for specific goals within the Basic Needs and Essential Services rights group. For a more complete description of these budget sub-categories within Basic Needs, see **Table 1** in the main paper.

***Figures 10a and 10b: Mortality ratio vs spending per capita on water and sanitation***

***Figures 11a and 11b: Mortality ratio vs spending per capita on education***

***Figures 12a and 12b: Mortality ratio vs spending per capita on shelter and infrastructure***

***Figures 13a and 13b: Mortality ratio vs spending per capita on non-food items***

***Figures 14a and 14b: Mortality ratio vs spending per capita on food security and nutrition***

***Figures 15a and 15b: Mortality ratio vs spending per capita on healthcare services***

**Secondary analysis using seemingly unrelated regression**

A secondary analysis was carried out on the spending and mortality data using seemingly unrelated regression to estimate a system of equations simultaneously with each equation corresponding to one budget category. This was performed using the budget categories corresponding to the eight major UNHCR objective groups represented in the *Focus* data. A ninth objective group, for “headquarters and regional support,” was not included because no spending was allocated to this budget category in the included PPGs. The STATA commands and raw output from this analysis and reproduced below.

The results using seemingly unrelated regression are similar to the results in the main paper using OLS regression, though not identical. With CMR as the outcome variable, spending for “fair protection processes and documentation” and for “logistics and operational support” show statistically significant associations with mortality at the α = 0.05 level, but spending for “external relations” no longer shows a significant association with mortality. Using U5MR as the outcome variable, spending on “fair protection processes and documentation” is a significant predictor of child mortality, but spending for “favorable protection environment” and “basic needs and essential services” no longer show a significant association with U5MR. Thus, we see that findings which were significant at the 1% level of significance for the series of separate OLS regressions are also significant with seemingly unrelated regression. Correlations with significance between the 1% and 5% level for the series of OLS regressions, however, are not statistically significant when seemingly unrelated regression is used.

**STATA commands and raw output**

***Using CMR as outcome variable, and eight budget categories as independent variables in system of eight regression equations***

. sureg (ln_cmrratio ln_favprot_pc_ppp ) (ln_cmrratio ln_fairprot_pc_ppp ) (ln_cmrratio ln_security_pc_ppp ) (ln_cmrratio ln_community_pc_ppp ) (ln_cmrratio ln_durable_pc_ppp ) (ln_cmrratio ln_extrel_pc_ppp ) (ln_cmrratio ln_logistics_pc_ppp ) (ln_cmrratio ln_basic_pc_ppp )

Seemingly unrelated regression

----------------------------------------------------------------------

Equation Obs Parms RMSE "R-sq" chi2 P

----------------------------------------------------------------------

ln_cmrratio 17 1 .5475011 0.0434 0.75 0.3867

2ln_cmrratio 17 1 .5080577 0.1763 5.22 0.0223

3ln_cmrratio 17 1 .5552399 0.0162 0.09 0.7695

4ln_cmrratio 17 1 .5586274 0.0041 0.01 0.9069

5ln_cmrratio 17 1 .5523739 0.0263 0.49 0.4829

6ln_cmrratio 17 1 .5407075 0.0670 1.15 0.2838

7ln_cmrratio 17 1 .5105966 0.1680 5.01 0.0252

8ln_cmrratio 17 1 .5420196 0.0624 0.76 0.3841

----------------------------------------------------------------------

-------------------------------------------------------------------------------------

| Coef. Std. Err. z P>|z| [95% Conf. Interval]

--------------------+----------------------------------------------------------------

ln_cmrratio |

ln_favprot_pc_ppp | -.0478335 .0552554 -0.87 0.387 -.1561321 .060465

_cons | -1.315519 .2266596 -5.80 0.000 -1.759763 -.8712741

--------------------+----------------------------------------------------------------

2ln_cmrratio |

ln_fairprot_pc_ppp | -.1290678 .0564897 -2.28 0.022 -.2397856 -.01835

_cons | -.9939411 .2383442 -4.17 0.000 -1.461087 -.5267952

--------------------+----------------------------------------------------------------

3ln_cmrratio |

ln_security_pc_ppp | -.0182588 .0623199 -0.29 0.770 -.1404036 .103886

_cons | -1.410569 .2699779 -5.22 0.000 -1.939716 -.8814222

--------------------+----------------------------------------------------------------

4ln_cmrratio |

ln_community_pc_ppp | -.0059792 .0511533 -0.12 0.907 -.1062378 .0942795

_cons | -1.459754 .2207994 -6.61 0.000 -1.892513 -1.026995

--------------------+----------------------------------------------------------------

5ln_cmrratio |

ln_durable_pc_ppp | -.046763 .0666529 -0.70 0.483 -.1774003 .0838742

_cons | -1.314326 .2701466 -4.87 0.000 -1.843804 -.7848483

--------------------+----------------------------------------------------------------

6ln_cmrratio |

ln_extrel_pc_ppp | -.062499 .058309 -1.07 0.284 -.1767826 .0517846

_cons | -1.305111 .2008224 -6.50 0.000 -1.698716 -.9115062

--------------------+----------------------------------------------------------------

7ln_cmrratio |

ln_logistics_pc_ppp | -.1006645 .0449583 -2.24 0.025 -.1887811 -.0125479

_cons | -1.002531 .2403306 -4.17 0.000 -1.473571 -.5314919

--------------------+----------------------------------------------------------------

8ln_cmrratio |

ln_basic_pc_ppp | -.0417788 .0480012 -0.87 0.384 -.1358595 .052302

_cons | -1.24687 .2931486 -4.25 0.000 -1.821431 -.6723097

-------------------------------------------------------------------------------------

***Using U5MR as outcome variable, and eight budget categories as independent variables in system of eight regression equations***

. sureg (ln_u5mrratio ln_favprot_pc_ppp ) (ln_u5mrratio ln_fairprot_pc_ppp ) (ln_u5mrratio ln_security_pc_ppp ) (ln_u5mrratio ln_community_pc_ppp ) (ln_u5mrratio ln_durable_pc_ppp ) (ln_u5mrratio ln_extrel_pc_ppp ) (ln_u5mrratio ln_logistics_pc_ppp ) (ln_u5mrratio ln_basic_pc_ppp )

Seemingly unrelated regression

----------------------------------------------------------------------

Equation Obs Parms RMSE "R-sq" chi2 P

----------------------------------------------------------------------

ln_u5mrratio 17 1 .6588017 0.0930 1.28 0.2575

2ln_u5mrra~o 17 1 .6235989 0.1873 4.29 0.0384

3ln_u5mrra~o 17 1 .6887688 0.0086 0.04 0.8333

4ln_u5mrra~o 17 1 .6915141 0.0007 0.00 0.9748

5ln_u5mrra~o 17 1 .6890017 0.0079 0.11 0.7445

6ln_u5mrra~o 17 1 .6785695 0.0378 0.45 0.5010

7ln_u5mrra~o 17 1 .6733989 0.0524 0.74 0.3910

8ln_u5mrra~o 17 1 .6647829 0.0765 1.09 0.2969

----------------------------------------------------------------------

-------------------------------------------------------------------------------------

| Coef. Std. Err. z P>|z| [95% Conf. Interval]

--------------------+----------------------------------------------------------------

ln_u5mrratio |

ln_favprot_pc_ppp | -.0992949 .0876957 -1.13 0.258 -.2711753 .0725855

_cons | -1.109957 .3330114 -3.33 0.001 -1.762648 -.4572668

--------------------+----------------------------------------------------------------

2ln_u5mrratio |

ln_fairprot_pc_ppp | -.1588004 .0767105 -2.07 0.038 -.3091502 -.0084506

_cons | -.8538456 .3160019 -2.70 0.007 -1.473198 -.2344933

--------------------+----------------------------------------------------------------

3ln_u5mrratio |

ln_security_pc_ppp | -.0138537 .0658192 -0.21 0.833 -.1428569 .1151496

_cons | -1.399463 .2980721 -4.70 0.000 -1.983674 -.8152526

--------------------+----------------------------------------------------------------

4ln_u5mrratio |

ln_community_pc_ppp | -.0016041 .0507312 -0.03 0.975 -.1010354 .0978272

_cons | -1.44699 .2409867 -6.00 0.000 -1.919316 -.9746652

--------------------+----------------------------------------------------------------

5ln_u5mrratio |

ln_durable_pc_ppp | -.0220902 .0677818 -0.33 0.744 -.1549401 .1107596

_cons | -1.374044 .2912272 -4.72 0.000 -1.944839 -.8032491

--------------------+----------------------------------------------------------------

6ln_u5mrratio |

ln_extrel_pc_ppp | -.0454544 .0675445 -0.67 0.501 -.1778391 .0869304

_cons | -1.324967 .2399889 -5.52 0.000 -1.795337 -.8545978

--------------------+----------------------------------------------------------------

7ln_u5mrratio |

ln_logistics_pc_ppp | -.0449618 .0524098 -0.86 0.391 -.1476831 .0577594

_cons | -1.239059 .2900547 -4.27 0.000 -1.807556 -.6705623

--------------------+----------------------------------------------------------------

8ln_u5mrratio |

ln_basic_pc_ppp | -.0613593 .0588277 -1.04 0.297 -.1766594 .0539409

_cons | -1.109349 .3589375 -3.09 0.002 -1.812853 -.4058443

-------------------------------------------------------------------------------------

.
